# Plasticity of NK cells in Cancer

**DOI:** 10.3389/fimmu.2022.888313

**Published:** 2022-05-10

**Authors:** Dillon Corvino, Ananthi Kumar, Tobias Bald

**Affiliations:** Tumor-Immunobiology, Institute for Experimental Oncology, University Hospital Bonn, Bonn, Germany

**Keywords:** Natural killer cell, Innate lymphoid cell (ILC), tissue-resident NK cell, decidual NK cell (dNK), intra-epithelial ILC1 (ieILC1), cancer immunotherapies, tumor microenvironment

## Abstract

Natural killer (NK) cells are crucial to various facets of human immunity and function through direct cytotoxicity or *via* orchestration of the broader immune response. NK cells exist across a wide range of functional and phenotypic identities. Murine and human studies have revealed that NK cells possess substantial plasticity and can alter their function and phenotype in response to external signals. NK cells also play a critical role in tumor immunity and form the basis for many emerging immunotherapeutic approaches. NK cells can directly target and lyse malignant cells with their inherent cytotoxic capabilities. In addition to direct targeting of malignant cells, certain subsets of NK cells can mediate antibody-dependent cellular cytotoxicity (ADCC) which is integral to some forms of immune checkpoint-blockade immunotherapy. Another important feature of various NK cell subsets is to co-ordinate anti-tumor immune responses by recruiting adaptive and innate leukocytes. However, given the diverse range of NK cell identities it is unsurprising that both pro-tumoral and anti-tumoral NK cell subsets have been described. Here, NK cell subsets have been shown to promote angiogenesis, drive inflammation and immune evasion in the tumor microenvironment. To date, the signals that drive tumor-infiltrating NK cells towards the acquisition of a pro- or anti-tumoral function are poorly understood. The notion of tumor microenvironment-driven NK cell plasticity has substantial implications for the development of NK-based immunotherapeutics. This review will highlight the current knowledge of NK cell plasticity pertaining to the tumor microenvironment. Additionally, this review will pose critical and relevant questions that need to be addressed by the field in coming years.

## Introduction

Innate Lymphoid cells (ILCs) were first described independently by various groups in 2008 and 2009. ILCs lack recombination-dependent antigen receptors present on adaptive B and T lymphocytes (1). Due to their cytotoxic and circulatory nature, natural killer (NK) cells are disparate from other members of the ILC family. As such, other ILC subsets are predominantly non-cytotoxic and tissue resident, principally providing cytokine support, inducing tissue repair and re-organization, and facilitating immune orchestration.

In 2013, the field first formalized ILC nomenclature and defined three distinct ILC groups. ILCs are grouped and distinguished by their principal transcription factor dependency and cytokine production. New insights into ILC development prompted a nomenclature revision and in 2018, the field recognized five distinct ILC groups (2, 3). Today, the ILC family includes NK cells, lymphoid tissue-inducer cells, and ILC groups 1, 2, and 3. ILCs have been implicated in various diseases and have been reported to contribute to both beneficial and detrimental outcomes (2).

In the context of cancer, ILCs can have either pro- or anti-tumoral effects (4). The mechanistic determinants of these effects are yet to be understood, but are largely dependent on the interplay between the particular ILC subset and the microenvironment of the diseased tissue (5). The described plasticity of ILCs poses added complexity. In response to environmental cues and pathological conditions, ILCs can switch phenotypes to acquire new or different functions (6). The triggers, mechanisms, and functional benefits or detriment of such plasticity are active areas of research. NK cells as part of the broader ILC family are no exception and have been described as a highly plastic cell type, with an ability to acquire numerous phenotypes and functional identities (7). These identities are dependent on tissue and disease status but are poorly understood in the context of tumor immunity.

Plasticity, differentiation, and development are three terms with at least partially overlapping meaning. It serves discussion to clearly define our use and meaning behind these terms. Within this text, plasticity is used as an overarching term which characterizes a cells ability to flexibly alter phenotype or function. As such, differentiation and developmental processes are also a variant of cellular plasticity. Therefore, all cells possess some degree of plasticity as even terminally differentiated leukocyte populations can usually alter their phenotype and become activated. Such an activation process can be considered cellular plasticity. Thus, the important biological questions are what is the degree of plasticity and towards which phenotypes can a cell progress. The terms, differentiation and development are more nuanced and refer to more directional processes or physiological processes occurring during development and homeostasis. This text will refrain from using the term differentiation and instead opt for defining processes by the broader term of plasticity.

NK cells are important for the control of primary tumor growth and mitigation of cancer metastasis (8–10). However, not all tumor-infiltrating NK cells are beneficial to patient prognosis. Various groups have observed pro-tumorigenic NK cell subsets infiltrating human tumors (11). These NK cell subsets are detrimental to disease outcomes and impact treatment prognosis but the origins of these cells are poorly understood. NK cells are increasingly being exploited as part of immunotherapeutic regimens, either through the administration of agents that aim to modulate NK function, or by adoptive transfer of large numbers of NK cells into patients (9). As such, it is important to understand how NK cell plasticity within the TME may shape NK cell responses. Understanding the mechanisms behind NK cell plasticity may be useful in improving the efficacy of current and future NK cell-based immunotherapeutics. This review will highlight our current knowledge of NK cell plasticity with respect to its impact within the TME and disease outcomes.

## Phenotype and Function of Circulatory NK Cells

Human NK cells are defined as lineage negative, CD56^+^ cells, where lineage markers include those used to define other major leukocyte populations including B cells, T cells, myeloid cells, and granulocytes (12). Importantly, CD56 is also expressed on a subset of myeloid cells and is dynamically regulated in NK cells upon activation (13, 14). As such, potentially *bona fide* NK cell populations can be CD56 negative. For example, CD56 negative NK cells are substantially elevated in patients with chronic viral infections such as HIV-1 or HCV (15). In addition, CD56 is unique to human NK cells and thus presents a challenge in translating and comparing findings across species (16).

**Figure 1:**
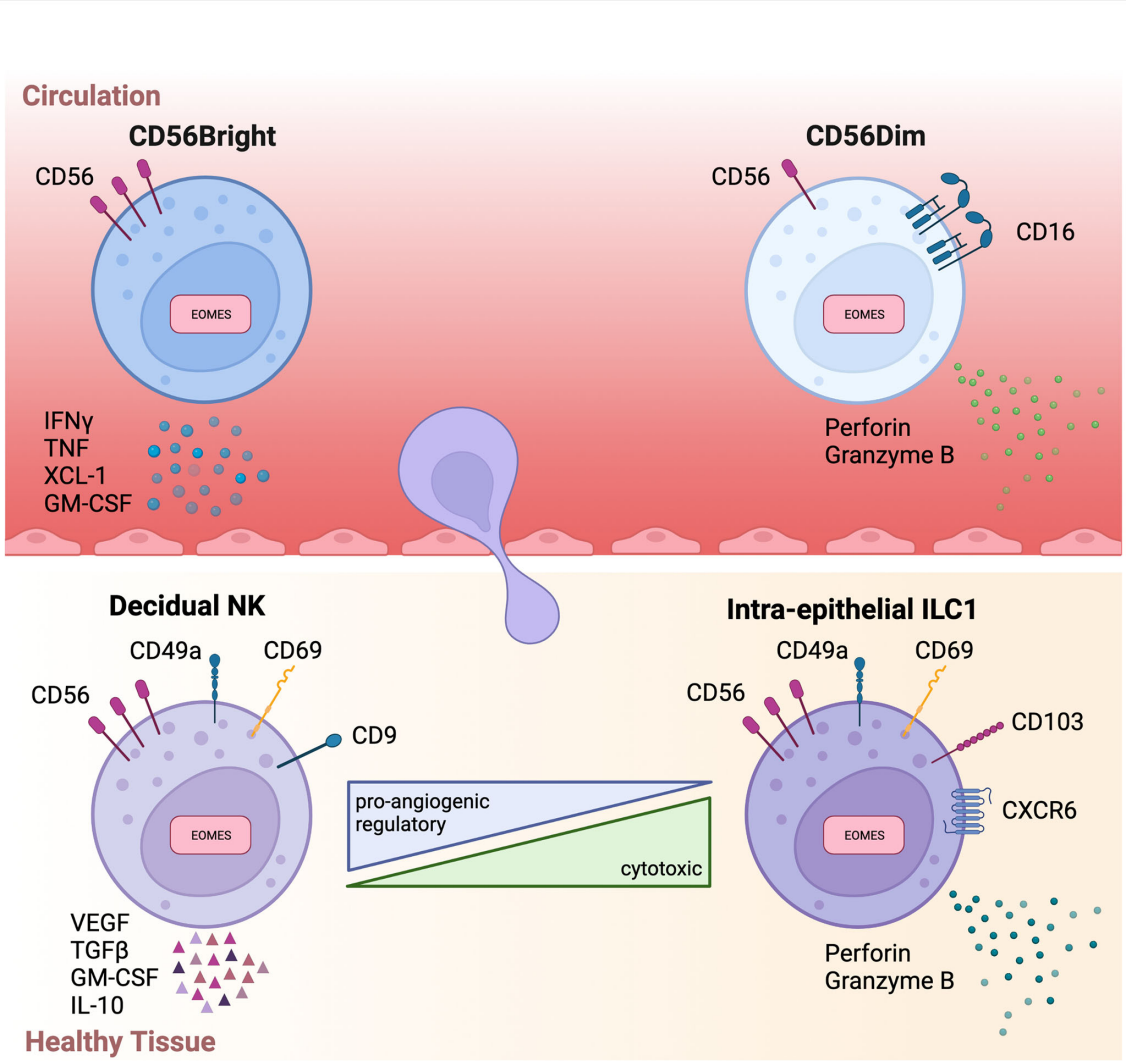
Overview of circulatory and tissue-resident NK phenotypes .Schematic summary highlighting key phenotype markers and functions of circulatory (above) and tissue-resident (below) NK cells. Interferon gamma (IFNγ), Tumor necrosis factor (TNF), Transforming growth factor beta (TGFβ), Granulocyte-macrophage colony-stimulating factor (GM-CSF), Vascular endothelial growth factor (VEGF).

In humans, two conventional NK (cNK) cell subsets have been identified based on their expression of the surface markers CD56 and CD16: CD56^Bright^ CD16^Dim/Negative^ (CD56Bright) NK cells and CD56^Dim^ CD16^Bright^ (CD56Dim) NK cells. These cNK subsets are both phenotypically and functionally distinct and have considerably disparate physiological functions and cellular distribution. CD56Bright cells are poorly represented in circulation, instead residing primarily in secondary lymphoid organs and tissues. Conversely, CD56Dim cells are dominant in peripheral blood, representing up to 95% of circulating NK cells. Functionally, CD56Bright cells are poorly cytotoxic, express low levels of killer immunoglobulin-like receptors (KIRs), and produce pro-inflammatory cytokines (17). Instead, CD56Bright cells are the predominant producers of immunoregulatory cytokines including interferon-gamma (IFNγ), tumor necrosis factor (TNF), IL-10, IL-13, and granulocyte-macrophage colony-stimulating factor (GM-CSF) (18) (**Figure 1**). Hence, CD56Bright cells are often referred to as pro-inflammatory NK cells. In contrast, CD56Dim cells are potently cytolytic and KIR^+^ (18). Importantly, CD56Dim cells, *via* the CD16 receptor, are responsible for antibody-dependent cellular cytotoxicity (ADCC), which in addition to their cytotoxicity, positions CD56Dim cells as a critical subset in cancer immunotherapy (19).

Relative to the CD56Dim population, CD56Bright cells are regarded as the more immature subset. Thus, CD56Bright cells are often described as the precursor to CD56Dim cells. Many lines of evidence support this developmental relationship between CD56Bright and CD56Dim cells. Namely, peripheral blood CD56Bright cells have longer telomeres than peripheral blood CD56Dim cells (20). Furthermore, *in vitro* experimentation has demonstrated that following certain stimuli, CD56Bright cells can acquire characteristics of CD56Dim cells (20, 21). However, this proposed linear developmental pathway from CD56Bright to CD56Dim cells is still controversial (22). Various observations are seemingly incompatible with a linear developmental trajectory. For example, the peripheral blood of patients with mutations in the transcription factor *GATA2*, are largely devoid of CD56Bright cells but retain the CD56Dim population (23). Additionally, lineage tracing experiments of hematopoietic stem cell reconstitution in rhesus macaques has revealed the macaque NK homologs CD56+CD16- (CD56Bright) and CD56-CD16+ (CD56Dim) arise from different progenitors (24, 25). How these data consolidate in a unified model of NK development is an active area of research. An additional layer of complexity is the dynamic regulation of CD56 and CD16 markers. Experimentation has shown that expression of CD56 and CD16 can be modulated by activation and stimulation. As such, engagement of CD56 with its ligand fibroblast growth factor receptor 1 can promote down-regulation of CD56 in CD56Bright cells (21). Whereas, stimulation of CD56Bright cells can induce upregulation of CD16 (20, 26). Conversely, *in vitro* stimulation can drive the upregulation of CD56 and downregulation of CD16 in CD56Dim cells (27). Thus, the dichotomy of CD56Bright and CD56Dim phenotypes breaks down under stimulatory conditions. As such, decerning cellular origin and developmental trajectory is particularly challenging in disease states. Further research employing surface marker agnostic technologies such as barcode-based lineage tracing, will be instrumental in delineating the developmental and pathological trajectories of NK cells.

Generally, NK cell infiltration and abundance are associated with positive prognosis and disease outcomes across multiple cancer entities (28). NK cells are integral to cancer immunosurveillance through multiple functions. NK cells can exert direct lysis of malignant cells *via* release of cytolytic granules or engagement of death receptors. It is unsurprising then that NK cells are crucial to limiting cancer metastasis as evidenced by the inverse correlation between their abundance and metastatic disease (8). Importantly, immune checkpoint-blockade efficacy has been associated with the presence of CD56 expression in post-treatment tumor biopsies of melanoma patients (29). Many studies, however, do not distinguish CD56Bright from CD56Dim cells, which have unique functions and may contribute to distinct outcomes. CD56Dim cells, for example, facilitate NK cell-mediated ADCC, which may be a key mechanism by which numerous monoclonal antibody treatments function (30). In contrast, increased frequencies of CD56Bright cells in malignant tissue and/or the peripheral blood of patients is associated with poor prognosis in melanoma (31, 32). The role of CD56Bright cells in disease prognosis is unclear and may be dependent upon various poorly defined variables. In bladder cancer patients, for example, intra-tumoral CD56Bright cells are associated with improved prognosis (33). This contradiction may in part be explained by NK cell functions that extend beyond direct killing of malignant cells. Specifically, NK cells are being increasingly recognized for their ability to recruit auxiliary cells and orchestrate the adaptive immune response (9). Murine studies have illustrated that NK cell-mediated recruitment of dendritic cells (DC) to the TME is important in controlling tumor growth and the efficacy of immune checkpoint blockade (34, 35). Critically, DC recruitment was facilitated by XCL1, which in humans is predominantly expressed by CD56Bright cells. Hence, although poorly cytotoxic, CD56Bright cells may be integral to anti-tumor immunity by orchestrating immune functions. To date, this perspective has been poorly evaluated and warrants further investigation.

## Tissue-Residency Nomenclature in Mice Versus Humans

Despite the similarities and parallels that can be drawn between mouse and human ILCs, there remains substantial interspecies differences. These differences are reflected in the varied nomenclature between species and inconsistencies with cellular phenotype and function. This notion is best illustrated with the comparison of NK cells and ILC1s across species.

NK cells and ILC1s are two highly intertwined subsets, both characterized by the expression of IFNγ and their dependence on the transcription factor TBET. In mice, these two subsets are recognised as distinct lineages arising from unique progenitor populations. Simplistically, within mice, these subsets are functionally and phenotypically delineated by two major factors—tissue-residency and cytotoxicity. Broadly, ILC1s are a tissue resident and non-cytotoxic population (2). As such, ILC1s are predominantly distinguished from NK cells in mice *via* the expression of tissue-residency markers, functionally, or by transcription factor expression (2). As ILC1s and NK cells arise through distinct lineages, NK cells are dependent on the transcription factor Eomesodermin (Eomes), whereas ILC1s are not. Hence, expression of Eomes is a principal marker identifying NK cells. Notably, mouse ILC1s in the salivary gland are identified as Eomes positive cells (36). In addition to Eomes, ILC1s in mice are defined by their expression of CD49a and CD49b. Mouse ILC1s are CD49a^+^CD49b^-^, whereas mouse NK cells are CD49a^-^CD49b^+^. This holds true for most tissues except salivary gland ILC1s and some populations in the small intestine, adipose tissue, and spleen where CD49a^+^CD49b^+^ populations have been described (36). Therefore, in mice the distinction between ILC1s and NK cells is relatively well defined.

Conversely, the distinction between human ILC1s and NK cells is unclear and remains controversial. At least three subsets of ILC1s have been described in humans: liver ILC1s, intra-epithelial ILC1s (ieILC1s), and CD127^+^ ILC1s. All three express the NK cell marker CD56, although there is some heterogenicity in CD56 expression in CD127^+^ ILC1s (36). In the seminal review defining the current nomenclature for both mouse and human ILC subsets, CD56 was identified as a unique NK cell marker (2). In addition, ieILC1s and CD127^+^ ILC1s both express EOMES to some degree (36). Hence, unlike in mice, ILC1s and NK cells are not easily distinguished by phenotypic markers. CD127^+^ ILC1s have also been reported in the circulation. Therefore, tissue retention does not serve as a viable distinguishing factor. In mice, ILC1s and NK cells can also be delineated by functional differences where ILC1s are generally non-cytotoxic and NK cells are cytotoxic. However, in humans, ieILC1s are reported as a cytotoxic subset and conversely, the CD56Bright subset of NK cells are non-cytotoxic (12, 36). Mass cytometry experiments have demonstrated that ieILC1s are more closely related to NK cells than helper ILC subsets (37). Similarly, sequencing data has revealed substantial overlap between human CD56Bright cells and mouse CD127^+^ helper ILC subsets (38). Specifically, CD56Bright cells and helper ILCs share expression of CD127, production of immunoregulatory cytokines, and expression of tissue residency markers such as CD49a and CD103 (7, 38, 39). Hence, distinguishing NK cells from ILC1s is difficult under the current nomenclature guidelines and functional definitions. Furthermore, the plasticity of NK cells and ILCs in inflamed tissues such as cancer adds to the complexity (6). Since 2018, the field has generated a wealth of data through high-dimensional technologies such as single cell sequencing and mass cytometry (7, 40, 41). This information could be used to refine the delineation of human NK cells and ILC1s and to standardize nomenclature.

## Phenotype and Function of Tissue-Resident NK Cells

In addition to the two conventional NK cell subsets, numerous phenotypically and transcriptionally unique NK cell populations have been described (39, 42). These distinct NK phenotypes predominantly represent various tissue-resident NK (trNK) populations, which inhabit a particular tissue microenvironment (12). Large sequencing and mass cytometry efforts have revealed that trNK phenotype and functionality is driven by their tissue of residence (7, 40). However, trNK subsets are closer aligned with one another than they are with circulatory NK cells.

**Figure 2:**
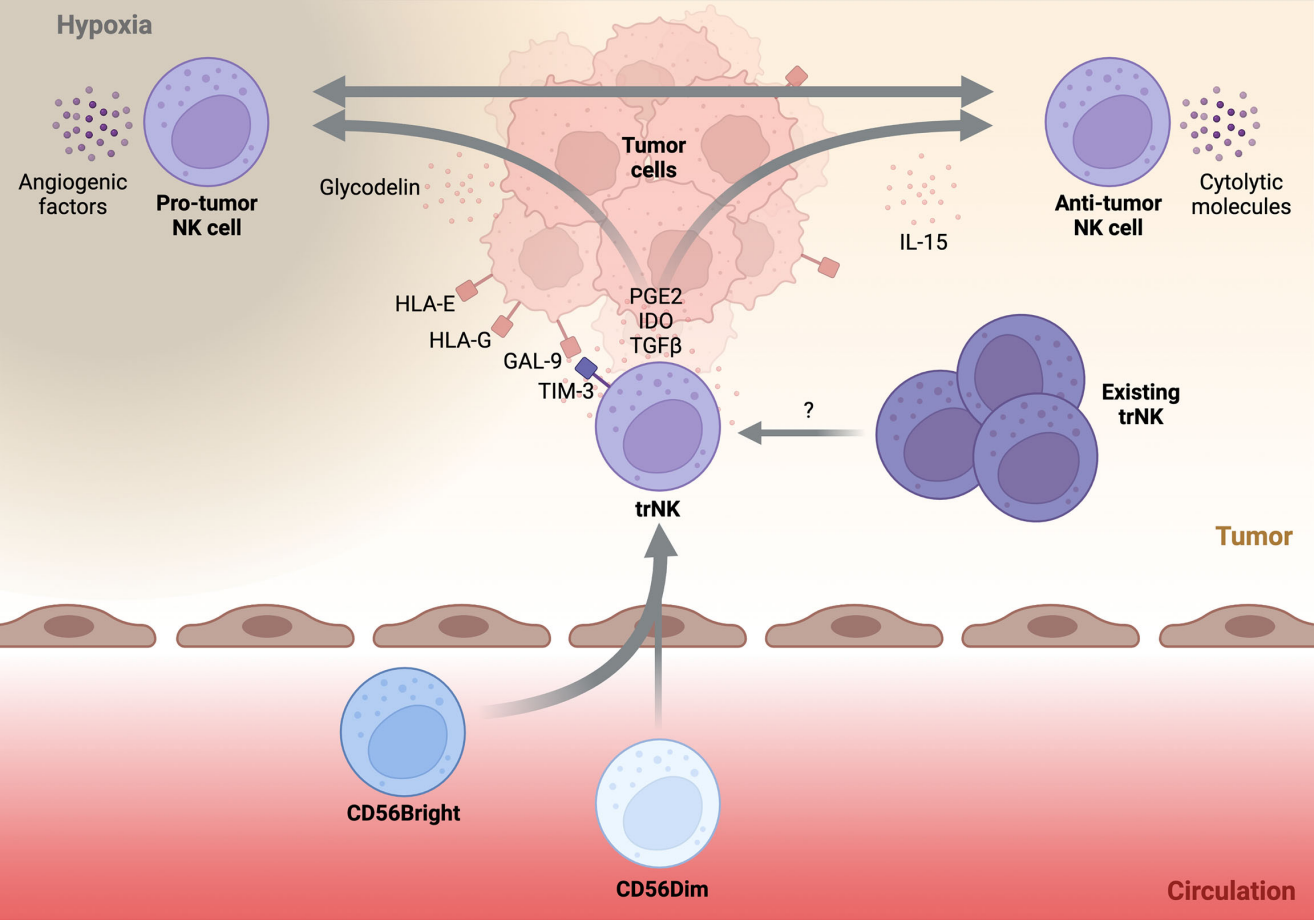
Schematic highlighting possible drivers and trajectories of tumor-infiltrating NK cells.A simplified schematic of the potential drivers and phenotypes of tumor-infiltrating NK cells. Circulatory NK cells (below) invade the tumor tissue (above) and acquire a tissue-resident NK (trNK) phenotype. Immunomodulatory factors within the tumor microenvironment such as, PGE2, IDO, and TGFβ help to solidify the trNK phenotype. Pre-existing trNK cells may also infiltrate the developing tumor and be directed towards various tumor-infiltrating NK phenotypes. Direct cell-cell contact between trNK cells and tumor tissue through non-classical HLA recognition or GAL-9-TIM-3 interaction may push trNK cells towards a pro-tumor phenotype. Additionally, secreted factors such as glycodelin or tissue hypoxia may promote pro-angiogenic properties of trNK cells. Alternatively, trNK cells may be driven towards an anti-tumor phenotype by stimulatory molecules such as IL-15. It is unknown if plasticity between the two trNK fates is possible and what factors drive this conversion. Transforming growth factor beta (TGFβ), Galectin-9 (GAL-9), Prostaglandin E2 (PGE2), Indoleamine-2,3-Dioxygenase (IDO).

In comparison to circulatory NK cells, trNK subsets are usually of a CD56^Bright^EOMES^Hi^TBET^Lo^ phenotype (12). EOMES expression in these trNK populations is yet another key difference between human and mouse. In mice, hepatic ILC1s are characteristically Eomes^-^ while the equivalent liver-resident NK population identified in humans are an EOMES^+^ population (43, 44). In addition to possessing a CD56^Bright^EOMES^Hi^TBET^lo^ phenotype, trNK cells also typically express a repertoire of tissue-retention molecules although the exact profile of tissue-retention molecules expressed is highly tissue dependent. Whilst the majority of trNK cells express CXCR6, CD69, and/or CD103 (12), there is no reliable marker that commonly identifies all variants of trNK cells. Despite being of a CD56Bright phenotype, trNK cells are distinct from circulatory CD56Bright cells in some key aspects. Firstly, trNK cells express tissue-residency markers not usually found on circulatory cells. Additionally, trNK cells are typically negative for CCR7 and CD62L, markers characteristically expressed by CD56Bright cells in circulation. Functionally, trNK cells are usually poorly cytotoxic and their production of proinflammatory cytokines is variable (12, 44, 45). Although numerous trNK subsets have been reported, two trNK subsets are frequently used to describe tumor-infiltrating NK cells: decidual NK (dNK) and ieILC1s (**Figure 2**).

The dNK cell is a specialized trNK subset physiologically found within the maternal *decidua basalis* during pregnancy. During pregnancy, dNK cells are the predominant leukocyte and can account for ~70% of total leukocytes within the tissue. Phenotypically, the majority of dNK cells are CD56^Bright^CD16^-^KIR^+^, in contrast to circulatory CD56Bright cells, which are KIR^-^. Additionally, dNK cells express both TBET and EOMES as well as an array of tissue-residency markers including CD9, CD49a, and CD69 (46). Physiologically, dNK cells are crucial to successful placentation through the orchestration of trophoblast invasion and endometrial vasculature remodelling. Although not entirely understood, dNK cells are thought to be important for the induction of immune tolerance and maintaining a tolerogenic environment at the maternal decidua (47). Given their specialised nature, it is no surprise that dNK cells have a unique functional phenotype. Most notably, dNK cells produce an array of pro-angiogenic factors such as vascular endothelial growth factor (VEGF), placental growth factor (PIGF), and angiopoietin-2 (Ang-2). In addition, dNK cells produce various immunoregulatory cytokines such as, transforming growth factor beta (TGFβ), GM-CSF, IL-8, and IL-10 (47), and are poorly cytolytic (46). Considering this, tumor-infiltrating NK cells displaying a dNK-like phenotype are unlikely to result in beneficial disease outcomes (**Figure 2**).

In contrast, ieILC1s represent the other extreme of possible trNK phenotypes that can be observed in the TME. Originally identified in tonsils and the intra-epithelial layer of the gut, ieILC1s are characterised as CD56^+^CD16^-^CD127^-^TBET^+^EOMES^+^ cells (48). Furthermore, ieILC1s can be identified by their expression of CXCR6 and the residency markers CD49a, CD103, CD69. ieILC1s have been observed in various mucosal and non-mucosal tissues (37). ieILC1 numbers remain relatively low outside of the tonsil or gut tissue at steady state although their abundance in pathological tissues can be markedly increased (37). The functional profile of ieILC1s is a stark contrast to dNK cells. Specifically, ieILC1s are cytotoxic, producing the cytolytic molecules granzyme B and perforin. Furthermore, upon stimulation with IL-15 or IL-18, ieILC1s can produce IFNγ at levels comparable to cNK cells (37) (**Figure 2**). In fact, a recent study found that tumor-infiltrating ieILC1-like cells in patients with head and neck squamous cell carcinoma (HNSCC) possess potent anti-tumoral function (49). Despite the existence of other trNK phenotypes, the characteristics of dNK and ieILC1 cells encompass those that also define tumor-infiltrating NK cells.

## ILC Plasticity

NK cells and the ILC subsets in general are often touted as having substantial phenotypic and functional plasticity (6). In response to environmental cues or disease perturbation, ILCs can alter their cellular identity to acquire a new functionality or phenotypes (6, 50). A situation where this occurs is cancer, which represents a major pathological disruption of homeostatic conditions. In cancer, tumor-infiltrating cells may become polarized to adopt a new phenotype and/or functional identity. Depending on the newly acquired identity, this process may be beneficial to the host and facilitate disease resolution. However, as highlighted in murine studies, NK cell plasticity may represent an immune evasion mechanism exploited by certain cancer entities (51, 52). Indeed, human tumor tissue hosts a range of NK phenotypes (53). Thus, tumor-infiltrating NK cells can adopt a spectrum of identities. As such, human NK cells also exhibit functional plasticity. However, the extent of plasticity, underlying mechanisms, and functional outcomes are poorly understood.

A thorough understanding of the breadth and scope of NK cell plasticity in the TME is critical to ongoing development of anti-cancer treatments. NK cell-based immunotherapeutics, be they cellular-based or antibody-mediated modulation of NK populations, are susceptible to the benefits and pitfalls of cellular plasticity. As NK cell-based immunotherapy becomes more mainstream, the field needs to consider their inherent potential for plasticity. A deeper understanding of NK plasticity will thus inform biomedical engineering strategies to promote and direct NK cells towards anti-tumoral fates as well as to maintain NK cells in a state that is optimal for anti-tumor immunity. The subsequent sections review current literature on NK cell plasticity and highlight its potential impact on tumor immunity and cancer immunotherapy.

### NK Cell Plasticity in Cancer

As previously highlighted, NK cells are often enriched in cancerous tissue. Broadly, this enrichment is associated with positive prognosis across multiple cancer entities (28). However, a more nuanced view reveals that the type of infiltrating NK can have a substantial impact on disease progression and patient prognosis. For example, enrichment of CD56Bright cells has been associated with poor prognosis (32).

Interestingly, tumor-infiltrating NK cells with unique phenotypes and functional properties have been observed in a number of different tumor types. These tumor-specific NK cell identities share significant similarities with certain trNK populations found in healthy individuals. As an example, various groups have described dNK-like cells in tumor tissue (54, 55). These cells align with a dNK definition in that they possess a CD56Bright phenotype, have decreased proinflammatory and cytolytic capacities, and most importantly produce pro-angiogenic factors. Indeed, dNK-like cells have been observed in non-small cell lung cancer (NSCLC) patients and strikingly, *in vitro* assays demonstrated that these dNK-like cells could induce the formation of capillary-like structures (55). Far from an isolated observation, similar dNK-like cells have been found infiltrating renal cell carcinoma, breast cancer, melanoma, colorectal cancer, and lung adenocarcinoma (54, 56–60). Furthermore, cells with a dNK-like phenotype have been observed in the peripheral blood of patients with prostate and colorectal cancer (58, 61). Tumor-infiltrating dNK-like cells and circulatory dNK-like cells have shown increased production of pro-angiogenic factors and an alteration of effector functions. However, the functional consequence of a dNK-like phenotype on anti-tumor immune responses is poorly investigated. Given the pro-angiogenic nature of these cells it is probable that enrichment of a dNK-like phenotype has a negative correlation with patient prognosis. There is some evidence to support this notion, for example, adoptive transfer of dNK cells into tumor-bearing mice results in a significant increase in tumor growth (62). Furthermore, in humans, VEGF within the tumor correlates with disease invasiveness, metastasis, and tumor progression (63).

In addition to the dNK-like phenotype, several studies have reported tumor-infiltrating NK cells with a trNK-like identity but with increased anti-tumor functions. These cells are sometimes described as ieILC1-like, given their similarities to physiological ieILC1 cells. In a murine tumor model, ieILC1 cells were demonstrated to have greater *in vitro* killing capacity than cNK cells. Furthermore, the authors demonstrated that ieILC1s were critical for tumor control (64). In humans, ieILC1s have been reported in colorectal and lung tumor tissue (37). However, the frequency of ieILC1s in colorectal tumor tissue was comparable to that of healthy colon tissue. In contrast, ieILC1s were considerably enriched in diseased lung in comparison to healthy lung mucosa (37). Recently, single cell RNA sequencing (scRNAseq) of ILC populations in HNSCC patients identified two subsets of ieILC1s (49). The authors reported these ieILC1 cells as expressing high levels of cytotoxic and proinflammatory genes. Subsequent *in vitro* assays with human ieILC1s revealed they had potent cytolytic activity against HNSCC tumor cells. In addition to the cytotoxic ieILC1 population, the authors also reported a dysfunctional NK cell subset. Trajectory inference analysis suggested that tumor-infiltrating NK cells could proceed down two distinct paths. One of these led to an ieILC1 phenotype and cytotoxicity whereas the other led to a dysfunctional phenotype (49). This study elegantly demonstrates a key question yet to be fully addressed: Upon tumor infiltration, what fate decisions do NK cells face? Furthermore, what governs these fate decisions and how reversible or permanent are the various cell trajectories? Unfortunately, much is still to be discovered; however, some pieces of the puzzle are beginning to come together. Experimental and observational evidence have revealed some of the critical signals driving these fate decisions.

### Drivers of NK Plasticity

What governs NK cell plasticity within the TME is a question crucial to the ongoing development of advantageous NK cell-based immunotherapeutics. This knowledge can be leveraged to promote tumor-infiltrating NK cells towards anti-tumoral phenotypes and mitigate against plasticity towards pro-tumoral phenotypes. However, much of the process determining NK cell fate and trajectory within the developing tumor tissue is poorly understood. In recent years new insights are beginning to build a picture of the multi-faceted signaling required to push NK cells towards certain intra-tumoral phenotypes. One such molecule highly implicated in tissue-residency and dysfunction is TGFβ (65, 66).

It is well established that TGFβ is responsible for imparting a tissue-resident signature on leukocyte populations. This is true for NK cells as well as other innate and adaptive immune subsets. For example, stimulation of cNK cells with TGFβ results in the rapid acquisition of tissue-residency markers (67, 68). Under physiological conditions, liver-derived TGFβ is important for maintaining the liver-resident NK phenotype (69). Similarly, TGFβ is found within the developing decidua during pregnancy and is essential for establishing the dNK phenotype (46). TGFβ is found in various cancer entities and is often associated with poorer disease outcomes (70–72). Within the TME, TGFβ exerts its function *via* numerous cell types and drives myriad responses (73). Broadly, the effect of TGFβ on NK cells can be characterized as immunosuppressive (74, 75). For example, tumor-derived TGFβ has been shown to mediate NK dysfunction in childhood B-acute lymphoblastic leukemia (76). With respect to NK plasticity, multiple groups have now demonstrated that TGFβ-signaling is a key driver in the acquisition of a trNK phenotype (51, 52, 68). However, not all functionalities observed in tumor-infiltrating NK populations can be explained by TGFβ alone.

Angiogenesis, for example, is a process predominantly initiated by tissue hypoxia, which induces the transcription factor hypoxia-inducible factor-1 (HIF-1). HIF-1 subsequently drives expression of pro-angiogenic factors (77). In line with this, *in vitro* culture of human NK cells has demonstrated that hypoxic conditions are essential for the induction of VEGF expression (68). Additionally, these *in vitro* experiments revealed that hypoxia-induced VEGF is exclusively observed in CD56Bright cells. The CD56Dim subset showed no capacity to produce VEGF following hypoxic treatment (68). The pro-angiogenic functions of NK cells have been demonstrated to be independent of TGFβ-mediated plasticity (78). Importantly, hypoxia is a common theme between the decidua and the TME. As such, hypoxia-induced signaling that reduces cytotoxicity and promotes pro-angiogenic functions are also encountered during tumor growth. In this manner, the developing tumor mass exploits intrinsic functional plasticity of NK cells and promotes a pro-tumorigenic phenotype.

The physiology of dNK cell development can provide insight into drivers that promote potentially detrimental intra-tumoral NK cell phenotypes especially given the many parallels that can be drawn between the immunoregulatory TME and the immunoregulatory maternal-fetal interface. Although many aspects of dNK cell physiology are unknown, their interaction with trophoblasts in the developing decidua is key to shaping dNK identity. Trophoblasts express a restricted HLA-I repertoire, predominantly HLA-C and the non-classical HLA-E and HLA-G. These non-classical HLA-I molecules are known to be atypically expressed within various cancer entities. Often, their expression is a negative prognostic marker. HLA-E and HLA-G can function in concert or isolation to inhibit activated NK cell function within the TME (79). In fact, signaling through the HLA-G receptor KIR2DL4 has been shown to induce NK cells to secrete pro-angiogenic factors and chemokines such as IL-8 (80). Additionally, trophoblasts express the regulatory molecules galactin-9 (Gal-9), a known ligand for the immunoinhibitory TIM-3 receptor. Gal-9 can be found elevated in human tumor tissue (81). Interaction between Gal-9 and TIM-3 is a known mechanism limiting NK cell cytotoxicity physiologically within the developing *decidua* and pathologically within the TME (82, 83). Interestingly, TGFβ stimulation has been shown to promote TIM-3 upregulation on cNK (83). Additionally, hypoxia can induce Gal-9 expression in human cancer (84). Taken together, certain signaling pathways within the TME may converge in feedback-loops and promote NK cell plasticity towards detrimental intra-tumoral phenotypes.

In addition to membrane-bound regulators, the decidual microenvironment and TME are rich in secreted soluble immunoregulatory molecules. Examples of these are the enzyme Indolemine-2,3-dioxygenase (IDO), the potent inflammatory mediator prostaglandin E2, and the glycoprotein glycodelin. IDO is expressed within most human tumors and the levels of IDO are associated with the loss of NK cell cytotoxicity (85, 86). Similarly, tumor-derived prostaglandin E2 in the TME has been shown to specifically modulate NK function and as a consequence disrupts the broader anti-tumoral immune response (87). Most intriguing, however, is the observation that glycodelin stimulation induces plasticity of peripheral blood CD56Bright cells towards a dNK-like phenotype. Glycodelin-treated CD56Bright cells up-regulated the dNK markers CD9 and CD49a and most critically, produced the pro-angiogenesis mediator VEGF. These effects were only observed in CD56Bright cells whereas CD56Dim cells showed no significant response to glycodelin treatment. The authors found that the glycodelin-induced response was mediated by CD62L, which is specifically expressed on circulating CD56Bright cells (88).

As previously described several immunomodulatory molecules present within the TME have been reported to impede NK cell function. However, the factors promoting plasticity of tumor-infiltrating NK cells towards an anti-tumoral phenotype are less defined. ieILC1s within the TME are a tumor-infiltrating NK cell phenotype that retains anti-tumoral cytotoxicity. Interestingly, ieILC1s have been demonstrated to be highly sensitive to IL-15 signaling. Researchers have shown using a murine model that IL-15 deficiency leads to the loss of ieILC1 cells, resulting in accelerated tumor growth (64). Similarly, *in vitro* assays with human NK cells revealed that IL-15 was important for the generation of ieILC1-like cells. Interestingly, trans-well assays showed that direct contact between NK cells and tumor cells was required for plasticity towards an ieILC1-like phenotype. Although, the tumor–NK signaling moiety that mediated NK to ieILC1-like plasticity within their model was not defined (49). In addition to IL-15, various other factors are abundant within the TME and may modulate NK function towards an anti-tumoral phenotype.

The TME is rich in damage associated molecular patterns (DAMPs), released from stressed and dying cells (89). Among other things, these DAMPs trigger production of type I interferons (IFN) from numerous innate subsets (90). Type I IFNs are known to augment NK functionality. Murine studies have demonstrated that type I IFN-signalling is important for NK anti-tumoral functions (91, 92). Similarly, type I IFN-signalling was found to be important for NK cytotoxicity in nasopharyngeal carcinoma patients (93). Furthermore, NK cells themselves can directly respond to various DAMPs *via* the toll-like receptor family (94). Additionally, the natural cytotoxicity receptors found on NK cells are suggested to function as DAMP receptors (95). As such, DAMPs within the TME are likely to modulate NK functionality, but how these signals modulate NK cell plasticity requires further investigation.

Nevertheless, this demonstrates that fate determination of tumor-infiltrating NK cells is a complex interplay between various secreted and membrane-bound factors. It is also plausible that plasticity towards pro- or anti-tumoral phenotypes is dependent on shifts in the balance between stimulatory and inhibitory signaling. However, there is little observational or experimental evidence to address this hypothesis. Further research is therefore urgently needed to ascertain the precise relationship between TME signals and resultant phenotype of tumor-infiltrating NK cells.

The relationship between circulatory CD56Bright cells and tissue-resident or tumor-infiltrating CD56Bright cells is poorly defined. Broad-tissue transcriptional studies have demonstrated that circulatory CD56Bright cells share key transcripts with tissue-resident CD56Bright populations (7). Furthermore, *in vitro* experimentation has shown sorted circulatory CD56Bright cells more rapidly acquire phenotypic traits and functions associated with tumor-infiltrating NK subsets. For example, IL-15 stimulation of CD56Bright cells drives ~80% of the population to adopt an ieILC1-like phenotype. In contrast, only ~40% of CD56Dim gained expression of ieILC1 markers CD49a and CD103 (49). Similarly, *in vitro* assays with sorted cNK populations have revealed that combined stimulation with IL-15 and TGFβ results in a greater proportion of CD56Bright cells acquiring tissue-residency markers (67, 96). Thus, it appears circulatory CD56Bright cells are particularly poised towards adopting a tissue-resident phenotype following certain environmental stimuli. In addition, there is some evidence to suggest that tumors may favour the recruitment of CD56Bright cells. For example, TGFβ has been shown to modulate the chemokine milieu towards expression of chemokines recognised by CD56Bright cells and reducing those recognised by circulatory CD56Dim cells. This concept is reviewed more comprehensively elsewhere (97–99). Thus, these data suggest tumor recruitment of circulatory CD56Bright cells may represent a mechanism for immune evasion. However, this hypothesis requires additional investigation. If validated, this would have implications for the development of immunotherapeutics and open additional avenues to direct NK infiltration of solid tumors.

Additionally, it should be noted that currently, the origins of the various tumor-infiltrating NK cell subsets are poorly understood. These tumor-infiltrating NK populations may also arise from *in situ* expansion of tissue-resident cells. Alternatively, tumor-infiltrating NK subsets may result from infiltration of circulatory NK populations and their subsequent plasticity towards the observed phenotypes (53). Currently, evidence supporting either model is largely observational. As previously outlined, given the dynamic regulation of key surface markers such as CD56 and CD16, these observations should be cautiously interpreted. In light of this, should tumor-infiltrating NK subsets arise from the infiltration of circulatory NK cells, the CD56Bright subset appears a strong candidate. Regardless, this concept is particularly challenging to study; as such, the field will need to utilise recent technological breakthroughs in order to dissect this process.

### Reversibility of trNK Phenotypes

There is mounting evidence that peripheral blood cNK cells can be polarized towards pro- or anti-tumoral phenotypes within the TME. However, it is unknown how stable these phenotypes are. What is the plastic potential of NK cells once they have infiltrated the tumor and acquired a particular phenotype? This is an important question to address as it will serve to inform immunotherapeutic reinvigoration strategies. Unfortunately, there is minimal research addressing “so-called” reverse polarization.

One study utilizing a genetic construct that linked *Eomes* expression to the *Tbet* locus, showed that ectopic expression of *Eomes* in murine ILC1s was sufficient to promote an NK cell phenotype in the modified ILC1 population. In principle, this highlights the potential reversibility of a tissue-resident phenotype. Similarly, two groups have independently shown that TRAIL^+^CD49b^-^ NK cells can convert into TRAIL^-^CD49b^+^ cells (100, 101). Again, this indicates that a tissue-resident phenotype may not be a terminal cell state and that trNK cells might retain considerable plasticity. Others on the other hand, have shown evidence that contradicts this. For example, adoptively transferred murine hepatic CD49b- NK cells, preferentially migrate to the liver and maintain their CD49b- phenotype (102). Similarly, Daussy et al. (103), demonstrated with the use of an *Eomes*-reporter mouse that the tissue-resident phenotype was stable. In this study, Daussy et al. (103), adoptively transferred Eomes positive or negative hepatic NK cells and assessed engrafting cell phenotypes in the liver. Under the current nomenclature, these hepatic Eomes^-^ NK cells would be classified as ILC1s. The authors found that Eomes^-^ NK cells preferentially engrafted the liver, however, they remained Eomes^-^ and did not give rise to Eomes^+^ cells (103). While it is unclear how the these seemingly contradictory datasets are aligned, these studies provide a basis for further investigation.

In humans, the evidence for reverse plasticity is scarce and limited to indications from *in vitro* experimentation. *Ex vivo* culture of liver-resident NK cells results in the gradual loss of their distinctive trNK phenotype (69). Similarly, *in vitro* culture of dNK cells stimulated with IL-15-containing media is sufficient to restore their defective cytotoxicity (68). Additionally, *in vitro* polarized dNK-like cells rapidly lose their pro-angiogenic functionality upon removal of dNK polarizing conditions (68). This highlights that continued environmental signals are required to maintain certain trNK phenotypes. It is important to note, however, that not all aspects of the dNK-like phenotype can be reversed. For example, tissue-residency markers remained high on polarized dNK-like cells long after polarizing conditions were removed (68). Hence, some phenotype traits may be more robustly imprinted than others or alternatively, require additional signaling to revert. It is thus unclear which trNK phenotypes are stable and which maintain plasticity. In a similar manner to the signals required to promote various tumor-infiltrating NK phenotypes, the signals necessary to revert these phenotypes remain poorly defined. Hence, additional research is required to understand the stability of these phenotypes and the mechanisms required to revert potentially detrimental phenotypes in the TME.

## NK Cell Exhaustion

Immunological exhaustion is a phenomenon characterized and extensively studied in the context of T-cell biology. Key hallmarks of immunological exhaustion include the reduction of cytotoxicity/cytokine production and the upregulation of immune inhibitory receptors (104, 105). Exhaustion functions as an immunological rheostat to limit unintended pathology occurring from sustained immunological responses.

Observational evidence has likened tumor-infiltrating NK phenotypes to immunological exhaustion. Indeed, tumor-infiltrating NK cells are often observed to have decreased cytolytic and cytokine capacities (106, 107). Additionally, tumor-infiltrating NK cells from various cancer entities are seen to upregulate inhibitory receptors such as PD-1 and TIM-3 (105). Furthermore, the inhibitory NK receptor NKG2A is increased in tumor-infiltrating NK cells from hepatocellular carcinoma patients compared with healthy control or peritumoral samples (108). Overall, these observations are consistent with the definition of immunological exhaustion.

Reconciliation of NK cell plasticity and exhaustion is a question yet to be addressed by the field. The phenotype of NK cell exhaustion has substantial overlap with aforementioned trNK phenotypes. Furthermore, akin to NK cell plasticity, immunosuppressive molecules elevated in the TME are heavily implicated in driving exhaustion. Thus, dysfunctional trNK phenotypes may reflect a state of exhaustion.

## Implications of NK Cell Plasticity on Cancer Immunotherapy

In recent years, cancer immunotherapy has resulted in improved disease prognosis. Through either the modulation of endogenous NK functionality *in vivo* or *via* transfer of exogenously generated/expanded NK cells, NK cell-based immunotherapeutics are at the forefront of cancer treatment development. Pre-clinical models and early-phase clinical trials have demonstrated the clinical potential of various NK cell-based immunotherapeutics (109, 110). However, as previously detailed, NK cells are a highly plastic population. This is especially true with respect to tumor-infiltrating NK cells, which encounter a barrage of immunosuppressive and immunomodulatory agents upon entry into the TME. In such instances, NK cell plasticity has the potential to push effective anti-tumoral subsets towards undesired and possibly even detrimental phenotypes *in vivo*. Hence, the development of NK-based immunotherapeutics needs to consider the inherent plasticity of NK cells as a potential factor limiting treatment efficacy. Strategies to mitigate and overcome undesired cellular phenotypes, and to promote and maintain desired cellular phenotypes can only be engineered with an understanding of the drivers, mechanisms, and implications of NK plasticity within the TME. In view of this, a number of groups have embarked on efforts that employing various strategies to promote efficacious phenotypes or limit detrimental cellular trajectories (111).

TGFβ signalling is an obvious pathway to target given the current knowledge of its detrimental impact on NK cell function. Accordingly, numerous researchers have invested into approaches designed to limit TGFβ signalling. A common approach is to genetically modify NK cells to express a dominant-negative TGFβ receptor that in effect, renders NK cells resistant to TGFβ-mediated signalling. As expected, these TGFβ-resistant NK cells show both higher *in vitro* and *in vivo* functionality against tumor targets. Consequently, TGFβ-resistant NK cells can more efficiently limit tumor proliferation and prevent disease metastasis than non-modified NK cells (112, 113). Instead of preventing TGFβ-signaling, others have used an alternative approach and engineered genetic constructs that invert TGFβ-signaling outcomes. By coupling stimulatory intracellular domains to the extracellular domain of the TGFβ receptor, researchers have derived stimulatory outcomes from TGFβ signalling. These modified NK cells have enhanced anti-tumor cytotoxicity and interestingly, these cells showed increased chemoattraction towards TGFβ-expressing tumor cells. Furthermore, these modified NK cells had improved *in vivo* persistence (114, 115). This last point is notable as NK cells are characteristically less persistent within patients than T-cells (116). These pre-clinical data demonstrate the potential therapeutic benefits of targeting TGFβ signalling. However, early phase clinical trials have shown that targeting the TGFβ pathway must be done in a precise manner. For example, broad-acting therapeutic agents targeting TGFβ bioavailability, interrupting ligand-receptor interactions, or disrupting the TGFβ signalling cascade have resulted in severe adverse events (117). Hence, to mitigate adverse events, it is important to focus disruption of TGFβ signalling to particular cells. Indeed, some early phase trials are evaluating the safety of targeting the TGFβ pathway in a more restricted fashion. For example, bi-specific molecules; which, couple a TGFβ trap with a PD-L1 antibody (118). Thus, restricting TGFβ sequestering to sites of high PD-L1 expression, such as, the TME. At the time of writing, there are only two registered trials looking to modulate the TGFβ pathway in conjunction with NK-based cellular therapy. These phase I and phase Ib trials (NCT04991870 and NCT05040568) are set to evaluate safety and activity of a cord blood-derived NK product for the treatment of recurrent glioblastoma or colorectal cancer patients with minimal residual disease, respectively. In both instances, the cord blood-derived NK product has been genetically engineered to lack the TGFβ receptor 2 in addition to a deletion of the glucocorticoid receptor NR3C1. Evidence has shown glucocorticoid signalling within NK cells results in inhibited cytotoxicity and cytokine production (119). As these trials are still in the recruitment (NCT05040568) or not yet recruiting (NCT04991870) phase, it will be some time before results are available.

Instead of blocking inhibitory signals such as those derived from TGFβ signalling, others are instead aiming to increase stimulatory signalling in NK cells. Common examples of this are methods targeting the IL-15 pathway, which is an attractive therapeutic target. Anti-CD19 chimeric antigen-receptor (CAR)-NK cells engineered to ectopically express IL-15 showed efficient killing of target cells *in vitro* and prolonged *in vivo* survival (120). Others have achieved promising results by simply infusing patients with IL-15 or an IL-15 superagonist (121, 122). Broadly, these engineering approaches were undertaken with the aim of improving NK functionality. This overlaps with, but is distinct from, engineering NK plasticity. Consequently, much of the phenotype and cellular identity of NK cells in these studies have not been evaluated in a manner that allows for the assessment of NK plasticity. Irrespective of this, the endpoint and primary aim is to improve therapeutic efficacy and these studies have demonstrated that modulating these pathways do lead to improvements in therapeutic efficacy. However, perhaps more can be gained by approaching this challenge with considerations for NK plasticity.

## Conclusions

NK cells can be found both in circulation and within various tissues. Physiologically, NK cells function both as nurturers where they orchestrate tissue remodeling and induce immune tolerance and alternatively, as first-line defense against infection and disease. This range in functionality is somewhat maintained in terminally differentiated populations. As a result, NK cells are said to possess great functional and phenotypic plasticity, with the ability to substantially alter their behavior and identity in response to environmental cues. Within tumor tissues, NK cells have been observed to have both pro- and anti-tumoral functions. The drivers of NK cell plasticity within the TME, however, are poorly understood. Although some key factors which act on tumor-infiltrating NK cells and promote certain phenotypes have been identified, the precise signaling required to decide NK cell fate towards pro- or anti-tumoral phenotypes is yet to be determined. Furthermore, the stability of these acquired phenotypes, and the drivers that facilitate reversal or a switch in acquired phenotypes of tumor-infiltrating NK cells is unknown. Perhaps insights can be garnered from the physiological development of various trNK populations under homeostatic conditions as these may represent pathways and processes exploited by pathological tissue to evade immune destruction. Understanding the complexity of NK cell plasticity within the TME could enable the field to design new and improved strategies to enhance NK-based immunotherapeutic approaches.

## Author Contributions

DC, AK, and TB wrote the manuscript and approved the final version.

## Funding

TB is funded by the Deutsche Forschungsgemeinschaft (DFG, German Research Foundation) under Germany’s Excellence Strategy – EXC2151–390873048.

## Conflict of Interest

The authors declare that the research was conducted in the absence of any commercial or financial relationships that could be construed as a potential conflict of interest.

## Publisher’s Note

All claims expressed in this article are solely those of the authors and do not necessarily represent those of their affiliated organizations, or those of the publisher, the editors and the reviewers. Any product that may be evaluated in this article, or claim that may be made by its manufacturer, is not guaranteed or endorsed by the publisher.
